# P-19. The Adjuvanted Recombinant Zoster Vaccine Is Effective in Preventing Herpes Zoster (HZ) Among Patients ≥ 50 Years of Age (YOA) With Selected Autoimmune Diseases in the United States: A Real-World Database Analysis (2018–2021)

**DOI:** 10.1093/ofid/ofae631.227

**Published:** 2025-01-29

**Authors:** Dagna Constenla, Germain Lonnet, Emmanuel Aris, Nathalie Servotte, Ramsanjay RK, Agnes Mwakingwe-Omar, Huifeng Yun

**Affiliations:** GSK, Rockville, Maryland; GSK, Rockville, Maryland; GSK, Rockville, Maryland; GSK, Rockville, Maryland; GSK, Rockville, Maryland; GSK, Rockville, MD, USA, Rockville, Maryland; GSK, Rockville, Maryland

## Abstract

**Background:**

The adjuvanted recombinant zoster vaccine (RZV) was approved by the US Food and Drug Administration (FDA) for HZ prevention in adults ≥50 YOA in 2017 and for adults ≥18 YOA who are at increased risk of HZ due to immunodeficiency or immunosuppression in 2021 (FDA, 2023. Shingrix. STN: 125614). Autoimmune disease (AID) patients are at increased risk of developing HZ compared to the general population. This real-world evidence (RWE) study assessed RZV vaccine effectiveness (VE) in patients ≥50 YOA with selected AIDs (rheumatoid arthritis, inflammatory bowel disease, systemic lupus erythematosus, multiple sclerosis, psoriasis and psoriatic arthritis).

**Figure:**
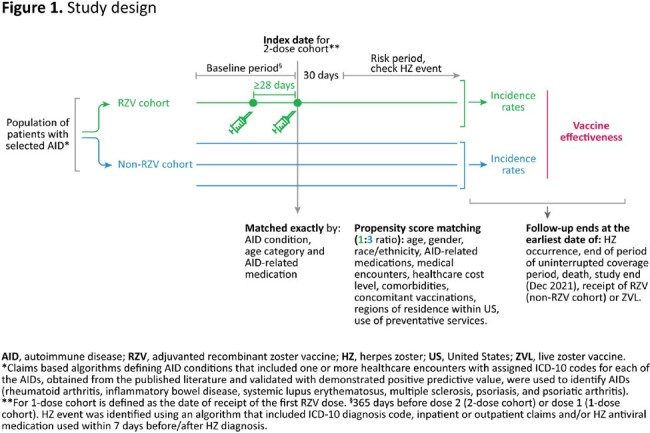

Study Design

**Methods:**

This retrospective matched cohort study was conducted in patients ≥50 YOA with selected AIDs using Optum’s de-identified Clinformatics Data Mart database from Jan 2018 to Dec 2021. Patients who received 2 doses of RZV (RZV 2-dose cohort) were matched (1:3) by age, medication category and propensity scores to unvaccinated counterparts (non-RZV cohort), as shown in **Figure 1**. Incidence rates (IRs) of HZ and VE were calculated overall, stratified by condition, age, gender, time interval between the 2 doses, and time since vaccination. Cox regression models were used to estimate hazard ratios (HRs). VE as % was calculated as (1−HR) × 100. A similar approach was used to evaluate VE for patients who received 1 RZV dose.

**Figure:**
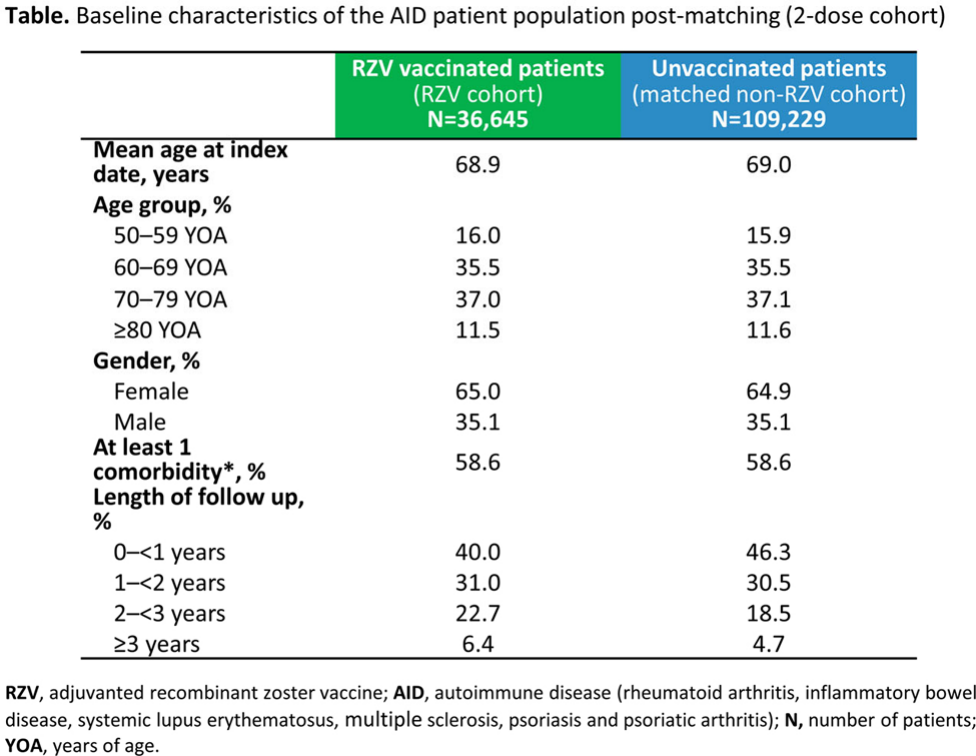

Baseline characteristics of the AID patient population post-matching (2-dose cohort)

**Results:**

We identified 36,645 RZV vaccinated and 109,229 matched non-RZV vaccinated AID patients in the 2-dose analysis (**Table**). Median follow-up was 1.29 (interquartile range [IQR]: 0.73–2.12) and 1.07 (IQR: 0.59–1.95) years for RZV and non-RZV cohorts. IR of HZ was 4.33/1,000 person-years (PY) in the RZV 2-dose cohort and 12.86/1,000 PY in the non-RZV cohort, resulting in an overall VE of 66.3% (95% confidence intervals [CIs]: 61.4–70.7) for AID patients. IRs and VE overall and stratified by each selected AID and variables are presented in **Figure 2**. Overall VE for AID patients who received 1 RZV dose was 58.3% (95% CIs: 45.8–68.0).

**Figure:**
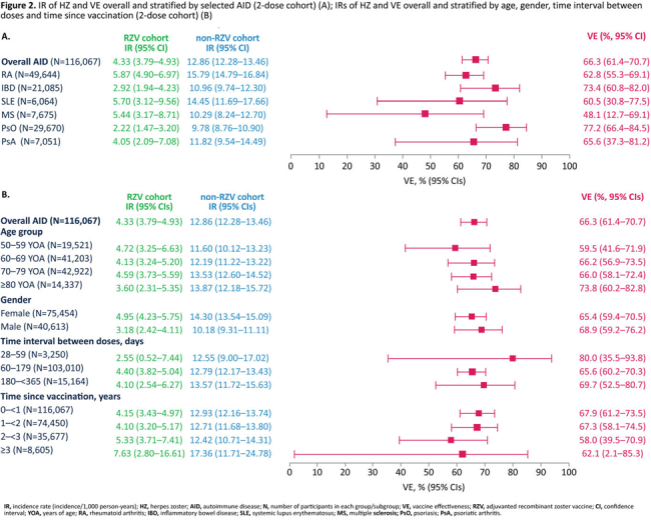

IRs of HZ and VE overall and stratified by selected AID (2-dose cohort) (A); IRs of HZ and VE overall and stratified by age, gender, time interval between doses and time since vaccination (2-dose cohort) (B)

**Conclusion:**

This analysis provides RWE that RZV vaccination is effective in preventing HZ in patients ≥50 YOA with selected AIDs. Our findings are consistent with the available literature on RZV effectiveness, which shows that RZV vaccination is beneficial to specific patient groups.

**Funding:** GSK

**Disclosures:**

**Dagna Constenla, PhD, MPA, MPhil, BA**, GSK: Employment|GSK: Stocks/Bonds (Private Company) **Germain Lonnet, MSc.**, GSK: Employment **Emmanuel Aris, PhD**, GSK: Employment|GSK: Stocks/Bonds (Public Company) **Nathalie Servotte, PhD**, GSK: Employment **Ramsanjay RK, B.Tech Mechanical Engineering**, GSK: Employment **Agnes Mwakingwe-Omar, MD, PhD**, GSK: Employed|GSK: Stocks/Bonds (Private Company) **Huifeng Yun, MD, PhD**, GSK: fulltime employee of GSK|GSK: Stocks/Bonds (Public Company)

